# Correlation Between Age and Body Composition Values Using Bioelectrical Impedance Analysis in Young Children

**DOI:** 10.7759/cureus.84192

**Published:** 2025-05-15

**Authors:** Naoki Hashizume, Saki Sakamoto, Motomu Yoshida, Tomohiro Kurahachi, Shiori Tsuruhisa, Naruki Higashidate, Daisuke Masui, Yoshinori Koga, Tatsuru Kaji

**Affiliations:** 1 Pediatric Surgery, Kurume University School of Medicine, Kurume, JPN

**Keywords:** bioelectrical impedance analyser, fat free mass, fat mass, phase angle, young children

## Abstract

Background and aim: Bioelectrical impedance analysis (BIA) is an attractive method for measuring the fat-free mass (FFM) and fat mass (FM) in adults. Although BIA is not typically performed in young children, previously published prediction equations for BIA in young children have been used recently. The present study evaluated the relationship between age, sex, and body composition in BIAs of young children.

Methods: This study is a retrospective cross-sectional analysis. All patients weighing >8 kg and 12-100 months old were included in the study. The body composition values in the BIA (FFM, FM, and phase angle (PhA)) were calculated. A multiple linear regression analysis for the body composition values in the BIA was used with the variables of age (months) and sex, as well as a constant.

Results: A total of 147 patients (86 boys and 61 girls) with a median age of 47 months were enrolled. Using multiple regression analyses, the FFM equation included the age, sex, and a constant (FFM (kg) = 0.128 × age + 0.391 × sex [male = 1, female = 0] + 6.378 [R^2^ = 0.911]). The equations for the FM and PhA included the age and a constant (FM (kg) =0.036 × age + 1.094 [R^2^ = 0.5068], PhA [°] = 0.021 × age + 3.533 [R^2^ = 0.482]).

Conclusions: The present study demonstrated that age and sex are significant predictors of body composition parameters in young children as measured by BIA. Specifically, FFM was strongly associated with both age and sex, while FM and PhA were moderately associated with age. The derived regression equations may serve as reference models for estimating body composition in pediatric populations using BIA.

## Introduction

Measurement of the body composition is crucial to assess a patient’s nutritional condition and can be estimated using a variety of techniques, such as cross-sectional imaging (computed tomography (CT) and magnetic resonance imaging (MRI)), dual energy X-ray absorptiometry (DXA), and a bioelectrical impedance analysis (BIA) [1]. CT and MRI are commonly used to determine anthropometric markers. Cross-sectional CT and MRI have been used to quantify skeletal muscle loss. The total psoas muscle area as evaluated by CT is an easy-to-measure marker of clinical nutrition [1-4]. However, CT is expensive and requires radiation exposure. While MRI has high accuracy for measuring skeletal muscle with the added benefit of no radiation exposure, it is quite costly and has a lengthy acquisition time, requiring sedation.

Bioelectrical impedance analysis (BIA) is an attractive method for identifying sarcopenia in adults and has gained popularity as a tool for predicting body composition [1]. It is a non-invasive, objective, direct, and quick (<90 s) method for determining nutritional and morbidity risks in patients. A BIA principally allows for the estimation of the components of the body structure, such as fat-free mass (FFM) and fat mass (FM), which are associated with the physical fitness of the human body composition. However, BIAs have not been widely used in young children because of inherent limitations, such as prediction errors.

The BIA technique relies on the availability of a prediction that is appropriate for the population being studied. For patients under six years, few tools are capable of measuring the body composition for prediction. However, several published prediction equations for FFM and FM in young children (two to five years old) using a BIA have been used [5-8]. Rush et al. developed the first prediction equation for a BIA using DXA for the FFM and FM in children two years old [5]. The regression model developed by Rush et al. included a multiethnic cohort of preschool children, including Asian participants [5]. Using this prediction equation, previous studies calculated the FFM and FM for children two to six years old [6-9]. However, there are few reports on prediction equations using age and sex for body composition values using a BIA in young children [7].

Recently, some studies have pointed out that BIA can serve as a good predictor to evaluate the disease, especially the BIA-derived phase angle (PhA). The PhA, which reflects cellularity and the cell membrane or cell function as indicative of the body composition for the BIA, is regarded as a composite measurement of tissue reactance and resistance of the BIA [10]. Lower PhA always represents a poor prognosis. A systematic review found PhA seemed to be a good indicator of mortality in many clinical situations [11]. PA has been linked with body water distribution, the extracellular water/total body water ratio (ECW/TBW), which is the standard parameter for evaluating body water balance and has also been reported as a useful parameter for determining the nutritional status and predicting an individual’s prognosis. ECW/TBW were calculated using the high and low frequency resistances of the BIA. However, studies on assessing the association between BIA measurement parameters and clinical outcomes in pediatric patients are lacking. The present study evaluated the relationship between age, sex, and body composition in BIA of young children.

## Materials and methods

Patients

All patients who were 12-100 months old, diagnosed with inguinal hernia, umbilical hernia, hydrocele, or undescended testicles, who underwent surgery at Kurume University Hospital between October 2013 and October 2018, were included in the study. The Z-scores of length/height were calculated according to the growth standard charts for Japanese children using the lambda-mu-sigma (LMS) method, with the closest LMS values for each measurement age used. Patients who had premature birth, low birth weight, and a height for age or body weight for age Z-score >2 or <-2 were excluded from the study. The patients underwent measurements for their subjective global assessment parameters, such as height, weight, and body mass index (BMI) (calculated as the weight (kg) divided by the height (m) squared (kg/m^2^). All patients were able to maintain a standing position and had their height and weight measured using a stadiometer and a weighing scale. All measurements were conducted under standardized conditions, with participants undergoing a four-hour fasting period and a two-hour restriction on both food and fluid intake prior to assessment. 

Bioelectrical impedance analysis (BIA)

For the BIA, an InBody S20 (Biospace, Tokyo, Japan) was used for the measurements in this study. Standardization of measurement in children used the details on the BIA protocol [12]. To prepare for the evaluations, patients were placed in the supine position in a thermoneutral environment at 28 °C. Their arms were positioned apart from their trunk, and their legs were positioned apart from each other. The BIA was performed with eight surface electrodes placed on the patient's thumb, middle finger, and both sides of each ankle. The time since the last meal was at least two hours. The time since the last void was not recorded. 

FFM and FM

The present study used the measured resistance at 50 kHz (R50) from a previously published empirically derived regression equation for FFM and FM developed using DXA in a cohort of young children by Rush et al. [6]. The reported equation is as follows: FFM (kg) = 0.367 × height (cm)^2^ / R50 + 0.188 × weight (kg) + 0.077 × height (cm) + 0.273 × sex (male = 1, female = 0) - 2.49. Furthermore, FM was computed from the body weight and FFM using the following equation: FM (kg) = weight (kg)- FFM (kg).

PhA

The PhA was determined at a single frequency (50 kHz) and calculated based on the sum of the impedance and reactance of the right arm, trunk, and right leg, using the following equation: PhA (°) = arctangent (reactance/resistance) × (180/π).

ECW/TBW

Resistance at 0 frequency represents ECW, and resistance at infinite frequency represents TBW. To calculate the close resistance, the ratio of the height squared to resistance determined at a low frequency (5 kHz) correlated most closely with the ECW using sodium bromide dilution as the standard of comparison. In contrast, the ratio of the height squared to resistance determined at a high frequency (500 kHz) correlated most closely with the TBW using deuterium oxide dilution as the standard of comparison. The ratio of the resistance at 500 kHz (R500) to the resistance at 5 kHz (R5) was directly correlated with the ECW/TBW. Previously, Cha et al. reported the equation as follows [13]: ECW/TBW = 0.757 × (R500 / R5) - 0.258.

Ethical approval

This study was performed after obtaining informed consent from the parents and approval from the Institutional Review Board at Kurume University School of Medicine (approval no. 17302).

Statistical analyses

The least squares mean was used to develop a multiple regression equation relating to body composition values. A stepwise regression approach analysis of the body composition for the BIA was used with the variables of age and sex, as well as a constant to verify a possible equation. All statistical analyses were performed using the JMP15 software package (SAS, Cary, NC, USA). The obtained data are expressed as the median (range)(25th and 75th interquartile range). To indicate statistical significance, p-values <0.05 were considered. The R^2^ value provides the proportion of the variance of an outcome variable that is explained by predictor variables in a multiple linear regression analysis model, such that a higher R^2^ indicates a better fit.

## Results

We reviewed 179 patients who underwent surgery at a single institution. Subsequently, 32 patients who had a premature birth, low birth weight, and height for age or body weight for age Z-score >2 or <-2 were excluded. A total of 147 patients (86 boys, 61 girls) who were a median 47 (12-100) months old were thus enrolled. The subjective global assessment parameters of the patients are shown in Table 1.

**Table 1:** The subjective global assessment parameters of the patients *The obtained data were expressed as the median (range) (25th and 75th interquartile range).

	All (n=147)	Boys (n=86)	Girls (n=61)
Age (months)*	47 (12-100) (27, 70)	35 (12-93) (23, 60)	57 (13-100) (41.5, 76)
12-23 months (n, %)	30 (20.4)	25 (29.1)	5 (8.2)
24-35 months (n, %)	27 (15.9)	19 (22.1)	8 (13.1)
36-59 months (n, %)	38 (25.9)	19 (22.1)	19 (31.1)
60-100 months (n, %)	52 (35.4)	23 (26.7)	29 (47.6)
Height (cm)*	97 (72-130) (86, 111)	91 (72-125) (84, 107)	103 (74-130) (92.5, 112.5)
Height (z-sore)*	–0.32 (-1.95-1.54) (-0.95, 0.19)	–0.32 (-1.95-1.54) (-0.92, 0.21)	–0.32 (-1.88-1.36) (-0.96, 0.09)
Weight (kg)*	15.0 (8.0-30.0) (12.8, 18.3)	14.0 (8.6-28.0) (11.1, 18.0)	17.0 (8.0-30.0) (13.0, 19.0)
Weight (z-sore)*	0.01 (-1.96-1.93) (-0.65, 0.60)	0.15 (-1.4-1.93) (-0.60, 0.64)	–0.30 (-1.96-1.48) ((-0.85, 0.48))
Body Mass Index (kg/m^2^) *	15.9 (13.4-19.7) (14.9,17.0)	15.4 (13.8-19.7) (15.4,17.3)	15.4 (13.4-16.8) (14.6,16.6)

Classified by age range, there were more girls who were 60-100 months old than 12-23 or 24-35 months old. The body composition of the patients for the BIA is shown in Table 2.

**Table 2:** Body composition for the BIA of the patients The obtained data were expressed as the median (range) (25th and 75th interquartile range). ECW/TBW: extracellular water/total body water, BIA: bioelectrical impedence analysis.

	All (n=147)	Boys (n=86)	Girls (n=61)
Fat-free mass (kg)	12.0 (7.2-22.1) (10.2, 15.0)	11.5 (7.7-20.0) (9.7, 14.8)	12.9 (7.3-22.1) (10.9, 15.3)
Fat mass (kg)	2.6 (0.7-8.1) (2.0,3.6)	2.4 (0.7-8.1) (1.8, 3.1)	2.9 (0.8-7.9) (2.3, 4.2)
Phase angle (°)	4.6 (2.7-6.4) (4.1,5.1)	4.5 (2.7-6.4) (3.9,4.9)	4.8 (3.3-6.0) (4.5,5.2)
ECW/TBW	0.388 (0.357-0.414) (0.383,0.395)	0.391 (0.366-0.414) (0.385,0.398)	0.386 (0.357-0.405) (0.382,0.391)

The multiple regression equation for body composition for the BIA using age and sex is shown in Table 3.

**Table 3:** A multiple regression equation for BIA parameters using the age and sex FFM: fat-free mass, FM: fat mass, ECW/TBW: extracellular water/total body water, BIA: bioelectrical impedence analysis

	Unstandardised coefficients		Standardised coefficients	t	p	95% confidence interbal for B	
model	B	Std error	beta			Lower bound	Upper bound
FFM							
(constant)	6.366	0.186		34.310	<0.0001	5.999	6.732
age	-0.382	0.083	5.628	-4.630	<0.0001	-0.545	-0.219
sex	0.128	0.003	-0.382	38.030	<0.0001	0.122	0.135
FM							
(constant)	1.172	0.171		6.850	<0.0001	0.834	1.511
age	0.035	0.003	1.539	11.260	<0.0001	0.029	0.041
sex	0.099	0.076	0.099	1.290	0.198	-0.051	0.249
Phase angle							
(constant)	3.559	0.105		33.960	<0.0001	3.352	3.766
age	0.021	0.002	0.909	10.870	<0.0001	0.017	0.024
sex	0.031	0.047	0.031	0.670	0.503	-0.060	0.123
ECW/TBW							
(constant)	0.398	0.002		247.750	<0.0001	0.395	0.402
age	-0.0001	0.000	0.000	-6.550	<0.0001	0.000	0.000
sex	-0.0006	0.001	0.000	-0.940	0.350	0.000	0.001

**Figure 1:**
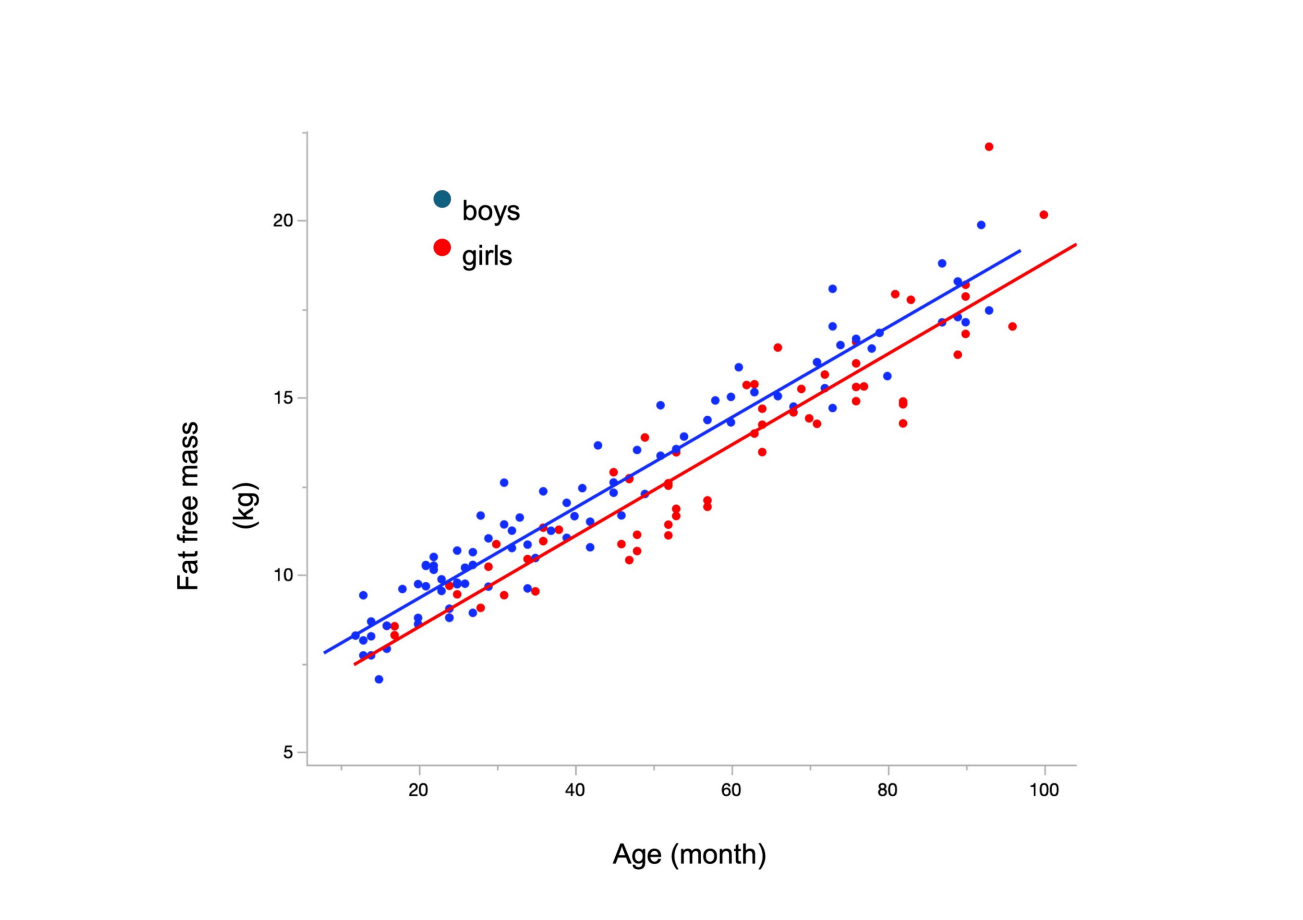
Scatter plots and fat-free mass based on the age and sex Fat-free mass (FFM) (kg) = 0.128 × age (months) + 0.391 × sex (male = 1, female = 0) + 6.378 (R2 = 0.911).

Using a multiple regression analysis, the FFM equation included the age, sex, and a constant. The prediction equation of the FFM was strongly correlated with age and sex, as follows: FFM (kg) = 0.128 × age (months) + 0.391 × sex (male = 1, female = 0) + 6.378 (R^2^ = 0.911) (Figure 1).

The FM, PhA, and ECW/TBW equations only included the age and a constant, as follows: FM (kg) = 0.036 × age (months) + 1.094 (R^2^ = 0.5068); PhA (°) = 0.021 × age (months) + 3.533 (R^2 ^= 0.482); and ECW/TBW = −0.0002 × age (months) + 0.399 (R^2^ = 0.2622).

## Discussion

The FFM was strongly correlated with age and sex in our cohort (R² = 0.911), suggesting that FFM in young children can be accurately predicted using these two variables. We developed a prediction equation for FFM based on BIA, which may serve as a standard parameter for body composition assessment in young children (Figure 1). To our knowledge, this is the first study to explore the relationship between age, sex, and BIA measurements specifically in this age group. Previous studies have shown limited availability of tools for assessing body composition in children under six years of age. Notably, Rush et al. developed one of the first BIA prediction equations for FFM and FM in two-year-old children using DXA as a reference standard [6-9]. Their equations have demonstrated good predictive performance, particularly in children over three years of age [7,9]. Given the age range of 12 to 100 months in our study, we adopted the prediction equation of Rush et al. as a reference model and found similar strong correlations with age and sex (R² = 0.911). These findings highlight the potential utility of BIA-based prediction equations as a baseline method for estimating FFM in young children. Such tools may prove especially valuable in clinical settings, allowing for the comparison of body composition among patients with different health conditions. Importantly, the effectiveness of this approach depends on the availability and suitability of prediction equations tailored to the specific population under study.

 FM, PhA, and ECW/TBW were moderately associated with age. PhA analyses are also useful for evaluating the nutritional status. The PhA has been evaluated in several previous BIA-related studies and is recognized as a useful factor for assessing the nutritional status of patients or predicting their prognosis [6]. BIA body composition measurements are sensitive to edema and the hydration status. Especially in children with liver, kidney, or cardiac diseases, where fluid overload is a problem, measurements have a high risk of error. However, the main advantage of using the PhA is that it can be applied even under unstable tissue hydration conditions, such as in cases with edema and ascites [13]. Xiong et al. reported that the PhA of 90-day survivors was significantly higher than that of non-survivors. Furthermore, an age-adjusted Spearman partial correlation analysis showed a weak negative correlation between the PhA and the duration of medical ventilation in young children [14].

A systematic review including five prospective studies found an association between lower PhA values at pediatric intensive-care unit admission and longer pediatric intensive-care unit and hospital lengths of stay, a longer duration of mechanical ventilation, septic shock, and increased mortality risk [15]. As with any biological marker, the PhA is influenced by the specific characteristics of each clinical population and may vary according to sex and age [16]. While the correlation was weak, the present study demonstrated important prognostic value of the correlation between the PhA and age with normal subjects in young children. This prediction equation may indeed be useful for monitoring the nutrition of patients with several diseases. PhA is rightly acknowledged as a prognostic marker, yet the low R² observed implies that its application in pediatric populations requires further clinical validation.

The ECW/TBW is the standard parameter for evaluating body water balance and has also been reported as a useful parameter for determining the nutritional status and predicting an individual’s prognosis. Furthermore, skeletal muscle mass (SMM) can be calculated based on the TBW value in the BIA [1]. In adult patients, a significant negative correlation was found between the ECW/TBW and the Alb and hemoglobin levels, and this value was consistently increased in subjects with a poor nutritional status. Moreover, the presence of a high ECW/TBW strongly suggests malnutrition and predicts a poor prognosis in critically ill patients. As a source of bias, young children have a higher water content than adolescents, and adults are similarly associated with a high ECW/TCW. In the present study, the equation revealed high ECW/TCW in young children. An overhydrated status with an ECW/TBW ≥0.4 leads to the overestimation of the SMM. Further studies are required.

DXA has become a widely used method for the measurement of body components in adults and children owing to its low radiation exposure, short scan time, low cost, and relative ease of use [17]. Appendicular lean tissue mass was extracted from whole-body DXA scans, and the whole-body SMM was calculated from the appendicular lean tissue mass using equations developed using MRI findings in children 5-14 years old [18]. However, to our knowledge, there have been no reports on the calculation of the SMM from whole-body DXA scans in this manner for children under five years old. At present, there are no accurate SMM equations using DXA or BIA in young children. The FFM is a component of the SMM that can identify cases of sarcopenia. Calculating the FFM leads to the evaluation of the SMM. However, there is a lack of consensus regarding the definition of sarcopenia in children [4]. Caution must be practiced when using FFM measurements to determine the SMM, as the FFM breakdown changes during childhood [3,19,20].

Several limitations associated with the present study warrant mention. First, this study had a small sample size, was conducted in a single region, and was a single-center study. This prediction model should be interpreted as preliminary and specific to the study sample. Thus, a further multicenter study with many age groups and healthy subjects not diagnosed with some diseases should be performed to verify the effectiveness of the BIA. One notable limitation of the present study is the absence of a healthy control group. While such a group would have allowed for more robust external comparisons and strengthened the generalizability of our findings, obtaining data from truly healthy children not undergoing any medical procedures posed significant ethical and logistical challenges. Second, the derived regression equation for the FFM was developed using DXA in a cohort of two-year-olds. In the present study, patients of a wide range of ages (12-100 months old) were analyzed. The possibility of an age-associated error, therefore, cannot be discounted. Third, the regression equation derived by Rush et al. was established using a cohort from New Zealand. Lyons-Reid reported that the inclusion of an additional ethnicity (Asian or non-Asian) would improve the prediction of FFM in young children [3]. While racial bias may contribute to systematic misestimation of certain physiological parameters in pediatric health assessments, there is currently no conclusive evidence in the literature to support the racial bias of young children. Third, the high R² for FFM indicates excellent model fit. However, lower R² values for FM, PhA, and ECW/TBW suggest that other unexplored variables may contribute significantly to body composition in young children. Finally, bladder voiding was not evaluated in the present study. Hydration misclassifications lead to an overestimation of the ECW/TBW ratio. The effect of bladder voiding has not been evaluated in any pediatric population, but in adults, bladder voiding is associated with a small measurement error [6].

## Conclusions

The present study demonstrated that age and sex are significant predictors of body composition parameters in young children as measured by BIA. Specifically, FFM was strongly associated with both age and sex, while FM, PhA, and ECW/TBW were moderately associated with age. The derived regression equations may serve as reference models for estimating body composition in pediatric populations using BIA. In the future, we will evaluate the utility of BIAs in critically ill young children using the predicted equations.
